# Supplementary material for: The Relationship between In Vitro and In Vivo Starch Digestion Kinetics of Breads Varying in Dietary Fibre
Source: Foods. 2020 Sep 22;9(9):1337. doi: 10.3390/foods9091337 (PMC7554867; doi:10.3390/foods9091337)
Supplement: Supplementary file 1 [file foods-09-01337-s001.pdf]

Supplementary information.

# *In vitro* starch digestion kinetics of breads varying in dietary fibre content and composition, compared with *in vivo* portal appearance of glucose in pigs

**Patricia E. Rojas-Bonzi [1], Cecilie Toft Vangsøe [1], Kirstine Lykke Nielsen [1], Helle Nygaard Lærke [1], Mette Skou Hedemann [1] and Knud Erik Bach Knudsen [1,*]**

[1] Aarhus University, Department of Animal Science, Tjele, Denmark
* Correspondence: knuderik.bachknudsen@anis.au.dk; Tel.: +45- 20647598

**Table S1.** In vitro cumulative starch hydrolysis expressed in % of dry starch, digestion rate k and asymptote digestion value.

| | Breads | | | | | | |
|---|---|---|---|---|---|---|---|
| **Time** | **WWB** | **WRB** | **WRBK** | AXB | BGB | SEM | *p*-value |
| 0 | 1.1 $^{b}$ | 0.7 $^{c}$ | 0.4 $^{d}$ | 3.2 $^{a}$ | 0.4 $^{d}$ | 0.04 | <0.0001 |
| 5 | 64.8 $^{a}$ | 57.6 $^{b}$ | 47.3 $^{c}$ | 59.2 $^{ab}$ | 45.2 $^{c}$ | 2.70 | 0.0002 |
| 10 | 65.6 $^{a}$ | 64.3 $^{ab}$ | 57.0 $^{bc}$ | 62.7 $^{ab}$ | 52.5 $^{c}$ | 2.49 | 0.0064 |
| 15 | 71.1 $^{ab}$ | 73.5 $^{a}$ | 67.9 $^{ab}$ | 64.0 $^{bc}$ | 58.7 $^{c}$ | 2.78 | 0.0128 |
| 30 | 85.6 $^{a}$ | 89.4 $^{a}$ | 82.2 $^{a}$ | 84.9 $^{a}$ | 72.2 $^{b}$ | 2.61 | 0.0061 |
| 60 | 91.8 | 89.5 | 85.1 | 95.8 | 89.9 | 2.5 | 0.46 |
| 120 | 94.6 | 90.0 | 92.6 | 96.5 | 92.7 | 4.27 | 0.84 |
| 180 | 94.8 | 93.4 | 95.2 | 98.9 | 93.5 | 3.04 | 0.74 |
| *k*, % hydr./min | 0.1595 $^{a}$ | 0.1462 $^{ab}$ | 0.1048 $^{ab}$ | 0.1000 $^{ab}$ | 0.0744 $^{b}$ | 0.02 | 0.06 |
| Asymptote, % | 89.8 | 89.6 | 89.5 | 94.4 | 90.2 | 3.1 | 0.75 |

WWB, white wheat bread; WRB, whole grain rye bread; WRBK, whole grain rye bread with kernels, AXB, wheat bread with arabinoxylan concentrate; BGB, wheat bread with β-glucan concentrate; $^{a,b,c}$ Mean values within a row with unlike superscript letters are significantly different ($p < 0.05$).
